# Thalassemias and Sickle Cell Diseases in Pregnancy: SITE Good Practice

**DOI:** 10.3390/jcm14030948

**Published:** 2025-02-01

**Authors:** Valeria Maria Pinto, Rosanna Cima, Rosario Di Maggio, Maria Livia Alga, Antonia Gigante, Filomena Longo, Anna Maria Pasanisi, Donatella Venturelli, Elena Cassinerio, Maddalena Casale, Raffaella Origa, Giovanni Zanconato, Gian Luca Forni, Lucia De Franceschi

**Affiliations:** 1 Centro della Microcitemia e Anemie Congenite e del Dismetabolismo del Ferro, Ente Ospedaliero Ospedali Galliera, 16128 Genova, Italy; dott.valeriapinto@gmail.com; 2 Dipartimento Scienze Umane, Università degli Studi di Verona, 37129 Verona, Italy; rosanna.cima@univr.it (R.C.); marialivia.alga@univr.it (M.L.A.); 3 Dipartimento di Ematologia e Malattie Rare, Azienda Ospedaliera Ospedali Riuniti Villa Sofia-Cervello, 90146 Palermo, Italy; rdm83@hotmail.it; 4 Società Italiana Talassemie ed Emoglobinopatie (SITE), 09121 Cagliari, Italy; segreteriascientifica@site-italia.org; 5 For Anemia Foundation ETS, 16100 Genova, Italy; 6 Day Hospital della Talassemia e delle Emoglobinopatie, Azienda Ospedaliero Universitaria S. Anna, 44124 Ferrara, Italy; filomena.longo@ospfe.it; 7 Centro della Microcitemia A. Quarta, Hematology Unit, A. Perrino Hospital, 72100 Brindisi, Italy; pasanisi.am@gmail.com; 8 Servizio Immunotrasfusionale, Azienda Ospedaliero-Universitaria di Modena, 41125 Modena, Italy; venturelli.donatella@aou.mo.it; 9 SS Emoglobinopatie, Disturbi Ereditari del Metabolismo e del Sistema Immunitario, SC Medicina ad Indirizzo Metabolico, Fondazione IRCCS Ca’ Granda—Ospedale Maggiore Policlinico, 20122 Milano, Italy; elena.cassinerio@policlinico.mi.it; 10 Dipartimento della Donna, del Bambino e di Chirurgia Generale e Specialistica, Università degli Studi della Campania “Luigi Vanvitelli”, 80138 Napoli, Italy; maddalena.casale@unicampania.it; 11 Dipartimento di Scienze Mediche, Università di Cagliari, SC Microcitemie e Anemie Rare Ospedale Microcitemico A. Cao, ASL Cagliari, 09047 Cagliari, Italy; raffaella.origa@unica.it; 12 Dipartimento di Scienze Chirurgiche, Odontostomatologiche e Materno-Infantili, Università degli Studi di Verona, 37129 Verona, Italy; giovanni.zanconato@univr.it; 13 Dipartimento di Ingegneria per la Medicina di Innovazione (DIMI), Università degli Studi di Verona e AOUI Verona, 37129 Verona, Italy; lucia.defranceschi@univr.it

**Keywords:** good practice, pregnancy, hemoglobinopathies, thalassemia, sickle cell disease, SCD, hemoglobin H disease

## Abstract

**Background:** Hereditary hemoglobin disorders are the most common globally distributed monogenic red cell diseases. The rights of women with thalassemia or sickle cell disease (SCD) to motherhood need to be protected by creating a roadmap to guide her, and her family network, along all the phases of the event. In fact, pregnancy in these vulnerable patients requires special attention and guidelines from the counseling stage (giving information about the special requirement and risks posed by their pregnancy with respect to the general population) the pre-conception stage, the early and mid-late pregnancy stage, to labor and lactation. The biocomplexity of these diseases requires a multidisciplinary team synergizing with gynecologists and obstetricians. In addition, the presence of a multicultural scenario requires healthcare workers to overcome stereotypes and adopt appropriate anthropological tools that might help them integrate the different cultural models of disease and motherhood. **Methods:** The Management Committee of the Society for Thalassemia and Hemoglobinopathies (SITE) selected and brought together a multidisciplinary and multiprofessional group made up of experts in hemoglobinopathies and experts in anthropology, flanked along with by experts with methodological and organizational expertise in order to create recommendations based on the integration of available scientific evidence together with expert opinion. **Results:** The panelists critically analyzed the literature, combining in a single document practices developed over several years of managing young women with hemoglobinopathies in a sensitive phase of their lives. **Conclusions:** This good practice document is the result of a collegial effort by Italian experts on hemoglobinopathies who are members of SITE. (SITE).

## 1. Introduction

Hereditary hemoglobin disorders (thalassemia syndromes, sickle cell diseases—SCD), named hemoglobinopathies, are the most widespread monogenic diseases worldwide [1,2,3]. In Italy, these diseases are endemic and were originally more frequently present in Sicily, the Po River delta and Sardinia. Nowadays, they are widespread throughout the national territory as a result of internal migration flows from south to north and the more recent international migration flows (Sub-Saharan Africa, Albania, Central and South America, Southeast Asia [1,2,3,4].

Advances in the clinical management and treatment of hemoglobinopathies have led to a marked improvement in the life expectancy and quality of life for patients suffering from thalassemia or sickle cell syndromes [5,6,7,8,9,10,11]. This has also led to a progressive increase in women with hemoglobinopathies who, having reached fertile age, express the desire for motherhood. In women with thalassemia and sickle cell syndrome, the management of pregnancy requires a multidisciplinary team, including experts in hemoglobinopathies, gynecologists, obstetricians, transfusionists, cardiologists, diabetologists, anesthesiologists/pain therapists, psychotherapists and other specialists, in case of organ complications related to the underlying pathology [12,13,14,15].

Furthermore, the presence of a multicultural panorama requires healthcare workers to overcome stereotypes and adopt appropriate anthropological tools that help them integrate the different cultural models of disease and motherhood [16,17]. In fact, studies conducted in French-speaking countries have highlighted how first-generation migrant pregnant women suffering from sickle cell disease present an increased risk of maternal death compared to second- or third-generation individuals of the same ethnicity and age residing in France [18,19].

In women with hemoglobinopathies, the transfusion regimen and iron chelation therapy change exactly in response to the pregnancy state and the pre-existing organ complications related to the hematological disease. In particular, the risk of alloimmunization and thromboembolism increases, with complications that can be fatal or highly disabling (e.g., delayed hemolytic transfusion reaction, pulmonary thromboembolism, cerebral ischemia) [12,14,20]. In women suffering from thalassemia syndrome, aspects related to the management of transfusion therapy and iron chelation therapy prevail, whereas in women with sickle cell syndrome, aspects related to obstetric-gynecological complications and management of acute events, like VOCs, prevail [12,13,14,15,18,21,22]. Preparation for childbirth, puerperium and breastfeeding are crucial moments that, once again, call for the need for a multidisciplinary team to accompany the patient on this journey.

The purpose of this “Good Practice” document is to describe the problems related to pregnancy in women with hemoglobinopathy and to provide recommendations for the management of every phase of gestation. Through the format of a pocketbook with abundant flowcharts, this document aims to answer questions that may arise in real life when dealing with these complex patients.

## 2. Materials and Methods

Considering the rarity of hemoglobinopathies, the Society for Thalassemia and Hemoglobinopathies (SITE) has undertaken a project aimed at systematically integrating available evidence with expert opinions in order to reach an adequate level of consensus on recommendations for clinical practice regarding pregnancy in women with hemoglobinopathy.

The Management Committee of SITE (LDF, GLF, VMP) selected and brought together a multidisciplinary and multiprofessional group made up of experts in hemoglobinopathies (EC, MC, RDM, FL, RO, AMP, DV) and anthropology (MLA, RC), along with experts with methodological and organizational expertise (AG), in order to create recommendations based on the integration of available scientific evidence together with expert opinion, with the aim of supporting clinicians in the decision-making process and improving the suitability of the therapies. JC, FM and SL assisted in preliminary phase of literature revision (see also Acknowledgement Section).

### 2.1. Searching Strategy and Selection Criteria

The panel of experts first divided the clinical problem into specific areas of interest and then, for each area, identified distinct scenarios and formulated the corresponding clinical questions. The panel chose to address the questions to the population with hemoglobinopathies in case the mechanism or characteristics of the object of the question were the same for thalassemia and sickle cell disease; instead, in case the mechanism or characteristics of the object of the question were different for thalassemia and sickle cell disease, the panel differentiated the question in the two specific populations. Transfusion-dependent thalassemia (TDT) and non-transfusion-dependent thalassemia (NTDT) were defined as previously reported [23,24].

**Figure 1:**
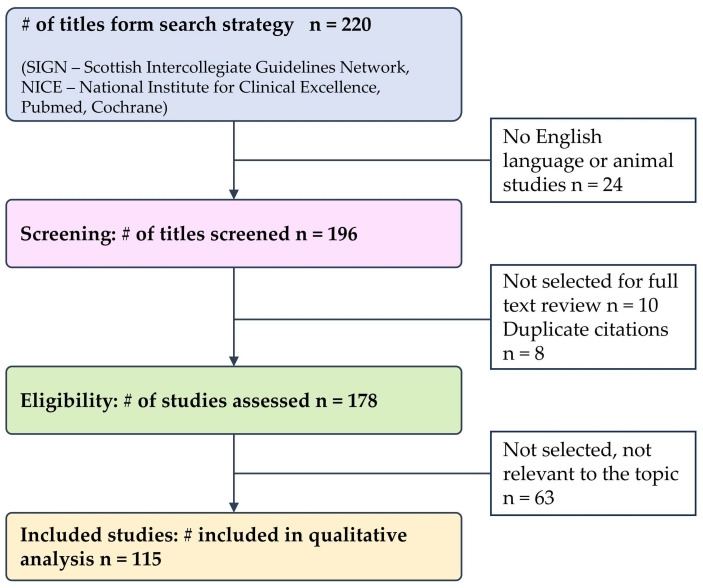
PRISMA flow diagram for screening process of the literature. #: number; PRISMA: Preferred Reporting Items for Systematic Reviews and Meta-Analyses.

The available evidence-based guidelines published between 1 January 2019 and 31 March 2024 have been identified, evaluated and selected; in consideration of the rarity of the pathologies, there was an extension from 5 to 7 years. Furthermore, in the absence of relevant scientific works in the identified time interval, we referred to registration or population studies, when available, up to 2005. Finally, in the absence of evidence-based data from prospective and randomized trials, the panel of authors had to refer to expert opinion (expert consensus) for many topics. The research was carried out in four main data sources: specific databases of guidelines; international health agencies producing guidelines; bibliographic databases referring to guidelines only; generalist databases (SIGN—Scottish Intercollegiate Guidelines Network, NICE—National Institute for Clinical Excellence, Pubmed, Cochrane). The search was completed manually and by questioning the panel experts about any “missing papers”. A critical evaluation of evidence in terms of quality, up-to-datedness and topics debated was carried out by the panel who deemed it necessary to integrate what had been selected with clinical studies focused on specific evidence for hemoglobinopathies. The literature search identified a total of 178 titles and abstracts. After a first evaluation—carried out based on abstracts—and a second one—based on full texts—a total of 115 reference documents were considered relevant (Figure 1).

After completing the literature review evaluation, the authors answered the questions, specifying the evidence used to support the answer. The authors presented and discussed what had been prepared during eight plenary meetings held in virtual mode between March 2022 and March 2024. Following the presentation of the answers, an informal process took place to reach consensus on the strength of the recommendations.

### 2.2. Grading Scheme

The following list shows the grading used in this good practice:

- Grade IA: strong recommendation based on strong evidence certainties and meta-analyses;
- Grade IB: strong recommendation based on strong evidence certainties;
- Grade IIA: strong recommendation based on moderate evidence certainties;
- Grade IIB: strong recommendation based on moderate/weak evidence certainties;
- Grade IIC: strong recommendation based on weak evidence certainties;
- Grade IIIA: conditional recommendation based on strong evidence certainties;
- Grade IIIB: conditional recommendation based on moderate evidence certainties;
- Grade IIIC: conditional recommendation based on weak evidence certainties;
- Grade IV: conditional recommendation based on expert indication.

Moreover, the answers by the panel of authors were also formulated based on common clinical experience, even in the absence of evidence or sufficient supporting evidence, on issues deemed relevant to clinical practice. No bias risk analyses, sensitivity analyses or variability assessments were performed.

The recommendations were written in a clear and unambiguous language. Where necessary, notes were added including information on limitations and conditions of applicability, as well as details on target populations, interventions, settings and outcomes. To improve wording, solve ambiguities, remove futile or potentially dangerous statements and suggest comments and criticalities, an editing process was performed. The final version of the document was forwarded, for an external review, to independent experts and representatives of patient associations to receive their comments and proposals for modification or supplementation. The comments received by revisers were considered by the authors’ panel, who replied to comments and decided which changes had to be made to the text based on such comments. In consideration of the continuous development of medical scientific knowledge and data that will gradually become available in literature, SITE will promptly update the document if new evidence becomes available or every 2 years. The methodology followed in the update will be the same used in the present version. Literature search shall begin from the date the present search was carried out. Once the GP document is deemed suitable for publication, it will be published on the SITE website; it will also be presented at the main conferences on hemoglobinopathies and will be translated and submitted for publication to an international peer-reviewed journal.

## 3. Results

### 3.1. Italian Law Protects Pregnancy of Women with Hemoglobinopathy

#### Question No 1 (Thalassemia, SCD): Is There a Law That Protects Pregnancy in Women with Hemoglobinopathy in Italy (Thalassemia or Sickle Cell Disease)? If So, Which Services Does It Provide Access to [25,26,27,28]?

Recommendation No 1: In addition to the pregnancy protection systems regulated by Italian law, the panel suggests informing women with hemoglobinopathy who wish to become pregnant that they can access specific services for their condition. During pregnancy, at the first visit (ideally within 10 weeks of amenorrhea), professionals should offer information and screening tests for hemoglobinopathies (sickle cell disease and thalassemia) to all women who have not previously received them. Family Counseling Centers can perform complete blood counts and diagnostic screening tests for hemoglobinopathies (e.g., high-performance liquid chromatography, HPLC). Blood count and dosage of hemoglobin (Hb) fractions (HbA2, Fetal Hb, Abnormal Hb) are among the specialist services provided by the Essential Levels of Care (LEA 2017) for the control of physiological pregnancy and are therefore free for all pregnant women and the partners of women who are heterozygous for hemoglobinopathies. In the case of pathological Hb, it is essential to contact an expert in hemoglobinopathies and start genetic counseling, at the same time carrying out a prompt evaluation of the partner, followed by a possible proposal of invasive prenatal diagnosis (e.g., amniocentesis or chorionic villus sampling). If the couple does not accept the invasive prenatal diagnosis, an evaluation of the hemoglobin defect in the newborn at birth is recommended.

### 3.2. Pregnancy: A Space for Multicultural Encounter

#### Question No 2 (Thalassemia, SCD): Should Pregnancy Offer Spaces for Multicultural Encounters? Which Tools Can Be Used [29,30,31,32,33,34,35,36]?

Recommendation No 2: The panel recommends considering pregnancy as a space for multicultural encounters and using relationship moments as tools for optimal birth support.

The tools useful for a favorable relationship between history and an understanding of pregnant women’s situation are as follows:

- Basis: Training in gender medicine:
  - Building a setting that allows the mother to express herself in her own language;
  - Identification of the language in which the woman is most proficient;
  - Use of the cultural linguistic mediator (CLM);
  - Medical history requires more time for cultural linguistic translation.
- Training of medical and healthcare personnel:
  - Be prepared to listen without judgement;
  - Direct questions aimed at knowing. There are different ways to represent pregnancy; consult the CLM on how to ask questions in order to build a relationship of trust between the doctor and patient;
  - “Culturally sensitive” listening to the experience of the disease;
  - Geopolitical knowledge of the patient’s areas of origin;
  - Knowing the patient’s community of reference (those who have a say, such as a family member, a spiritual guide, …).

### 3.3. Management of Preconception Period in Patients with Hemoglobinopathies

#### 3.3.1. Question No 3 (Thalassemia): Should a Woman with Thalassemia Who Wishes to Become Pregnant Undergo Specific Checks in Addition to Those Required for Healthy Women [37]?

**Figure 2:**
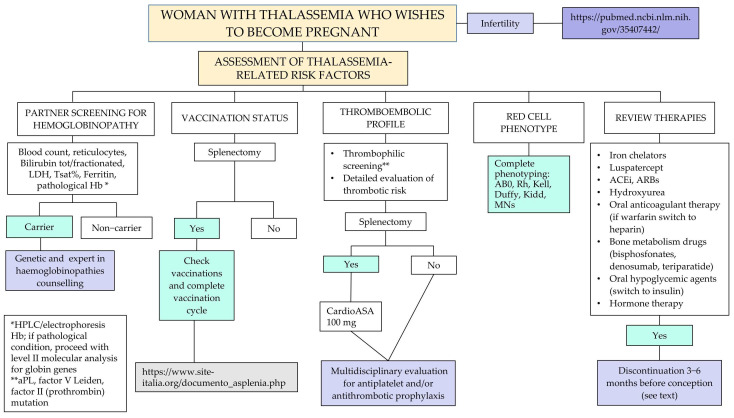
Flowchart 1.1. Assessment of risk factors in thalassemic women. LDH: lactate dehydrogenase; Tsat%: transferrin saturation percentage; Hb: hemoglobin; HPLC: high-performance liquid chromatography; aPL: antiphospholipid antibody; ASA: acetylsalicylic acid; ACEi: angiotensin-converting enzyme inhibitor; ARBs: angiotensin receptor blockers.

**Figure 3:**
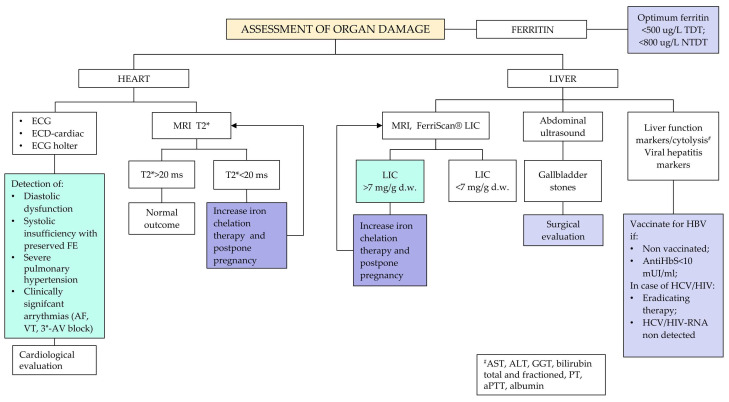
Flowchart 1.2. Assessment of organ damage in thalassemic women. TDT: transfusion-dependent thalassemia; NTDT: non-transfusion-dependent thalassemia; ECG: electrocardiogram; ECD: cardiac echocolordoppler; EF: ejection fraction; AF: atrial fibrillation; VT: ventricular tachycardia; 3rd-AV block: atrioventricular block third degree; MRI: magnetic resonance imaging; T2*: T2 star; ms: millisecond; LIC: liver iron concentration; d.w.: dry weight; HBV: hepatitis B virus; antiHBs: hepatitis B surface antibody; HCV: hepatitis C virus; HIV: human immunodeficiency virus; RNA: ribonucleic acid; AST: aspartate transaminase; ALT: alanine transaminase; GGT: gamma-glutamyl transferase; PT: prothrombin time; aPTT: activated partial thromboplastin time.

**Figure 4:**
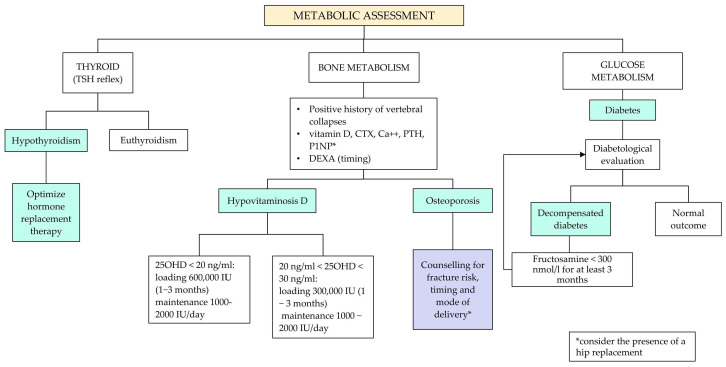
Flowchart 1.3. Metabolic assessment in thalassemic women. TSH: thyroid-stimulating hormone; CTX: C-terminal telopeptide; Ca++: calcium; PTH: parathyroid hormone; P1NP: procollagen 1 intact N-terminal propeptide; DEXA: dual-energy X-ray absorptiometry; 25OHD: 25-Hydroxyvitamin D3; IU: international unit.

Recommendation No 3: For all women with thalassemia who wish to become pregnant, the panel recommends, in addition to what is provided for healthy women, the following:

- Anamnestic evaluation of vaccination status, thromboembolic profile, immuno-hematological profile and current pharmacological therapy (Figure 2, Table S1). The panel recommends discontinuing pharmacological therapy three months before attempting pregnancy. Accurate counseling on the risk related to the interruption of their treatment should be carried out, followed by the request for patient consent before proceeding. The following drugs should be withdrawn: iron chelators, luspatercept, hydroxyurea (HU, see also Recommendation No 12, note on the use of hydroxyurea in pregnancy). For oral anticoagulants, the patient will have to shift from oral anticoagulant therapy (warfarin, direct oral anticoagulants—DOAC) to therapy with low molecular weight heparin (LMWH); for bone metabolism drugs, discontinuation is indicated three months before for bisphosphonates, while for other therapeutic strategies (e.g., denosumab, teriparatide), an evaluation with the bone metabolism expert is indicated. Concerning antihypertensive molecules such as angiotensin-converting enzyme inhibitors (ACEi) and angiotensin receptor blockers (ARBs), patients need to be switched to other antihypertensive medications not long before pregnancy in agreement with the reference gynecologist/obstetrician;
- Organ damage assessment with a focus on the heart and liver (Figure 3);
- Endocrine-metabolic assessment including thyroid and glucose pattern and bone status (Figure 4).

Cardiac performance should be assessed in all women with electrocardiogram (ECG), cardiac echocolordoppler (ECD), Holter ECG and cardiological evaluation (where available with a cardiologist with expertise in the management of thalassemia) (Figure 3).

Liver function should be assessed in all women, and testing for toxoplasmosis, other infections, rubeola, cytomegalovirus and herpes (TORCH) should be combined with the assessment of serological markers for the different forms of viral hepatitis and human immunodeficiency virus (HIV). In particular, an abdominal ultrasound should be performed on all women to evaluate the presence of gallbladder stones, liver cirrhosis and splenomegaly. Perform a Fibroscan, evaluating case by case (Figure 3)

The panel recommends the following:

- Women with adequate iron chelation (liver iron concentration—LIC < 7 mg/dL, T2* > 20 ms): 1 to 3 months before conception, discontinue oral iron chelation therapies (and therefore the introduction of deferoxamine) since there are no conclusive data on the teratogenicity of oral iron chelators. Moreover, there is a greater number of pregnancies described with no fetal malformation outcomes in women treated with deferoxamine in the weeks preceding pregnancy or during pregnancy before it was identified.
- Women with NOT adequate iron chelation (LIC > 7 mg/dL, T2* < 20 ms): Consider possible intensive iron chelation (combination therapy of deferiprone and deferoxamine or, in case of intolerance or history of unacceptable side effects, other associations between iron chelators or monotherapies at the highest tolerated dose) since iron overload affects maternal-fetal prognosis (Figure 3);
- Women with beta-thalassemia NEVER transfused (e.g., non-transfusion-dependent thalassemia—NTDT): Perform complete characterization of red cell phenotype with counseling on the increased risk of onset of immune anemia and its impact on maternal-fetal health (Figure 2);
- Women with endocrine-metabolic pathology:
  - Thyroid function: Maintaining a correct concentration of thyroid hormones in circulation is indeed of fundamental importance to guarantee the correct development of the fetus, especially as far as the central nervous system is concerned, thus avoiding the onset of cognitive impairments. Moreover, if hyperthyroidism is not treated, the likelihood of maternal and fetal complications increases.
  - Glucose metabolism: Glucose intolerance, gestational diabetes, diabetes. The woman should undergo an examination by a diabetes specialist since good glycemic control is essential for maternal-fetal health. Additionally, similar to what is recommended for non-thalassemic women with diabetes, pregnancy should not be undertaken in the presence of poor glycemic control (main endocrinological complications in hemoglobinopathies: good clinical practice by the Società Italiana Talassemie ed Emoglobinopatie). A discontinuation of the oral antidiabetic drug with the shift to insulin is recommended.

Pregnancy planning is an important aspect to be enhanced in the setting of hemoglobinopathies. Pre-pregnancy evaluation also involves the partner. If the partner is a hemoglobinopathy carrier, it is recommended to offer the couple genetic counseling and hemoglobinopathy expert counseling (Figure 2). These couples should be informed about the aspects of the disease including the possibility of prenatal diagnosis, the risks associated with the procedure, and the possibility of medical abortion within the 22nd week. Pregnancy is often already underway at the time of the first evaluation and counseling should be implemented as soon as possible.

Strength of the Recommendation: Strong based on moderate/weak evidence certainties

Degree of evidence: IIb

Panel agreement: Full agreement

#### 3.3.2. Question No 4 (Thalassemia): In Which Clinical Conditions Is It Not Advisable for a Woman with Thalassemia to Become Pregnant [12,21,38,39,40,41,42]?

Recommendation No 4: The panel suggests not to become pregnant in the following conditions:

- Liver iron overload: LIC > 7 mg/g;
- Heart iron overload: T2* < 20 ms;
- Chronic hepatitis C virus (HCV) infection: HCV-RNA detection;
- Hypothyroidism: if suboptimal replacement therapy;
- Diabetes: fructosamine concentration > 300 nmol/L;
- Systolic-diastolic dysfunction, severe pulmonary hypertension or clinically significant arrhythmias;
- Severe alloimmunization with non-transfusable profile.

In the event that the woman with beta-thalassemia during the pre-pregnancy evaluation presents one or more conditions that could contraindicate pregnancy due to high maternal or embryo-fetal risk, it is recommended that the pathology expert conducts an informational interview with the patient and her partner, taking advantage of the contribution of the other specialists involved, with a multidisciplinary approach, which is essential for the maternal-fetal outcome.

Strength of the Recommendation: Strong based on moderate/weak evidence certainties

Degree of evidence: IIb

Panel agreement: Full agreement

#### 3.3.3. Question No 5: Are There Any Peculiarities in Pregnancy of Women with Hemoglobin H Disease [43,44,45,46,47]?

Recommendation No 5: The panel makes the following recommendations:

- Do NOT start antiplatelet/antithrombotic prophylaxis in the absence of other identified thrombotic risk factors;
- Maintain hemoglobin levels ≥ 10 g/dL by constantly monitoring fetal growth;
- Follow the indications regarding supplementation (folic acid, vitamin D) in pregnant thalassemic women.

Hemoglobin H disease generally has a limited impact on pregnancy, and most experiences reported in the literature highlight the absence of major complications.

The panel identifies two groups at increased risk of complications:

- Non-deletion hemoglobin H: pre-eclampsia and congestive heart failure.
- Hemoglobin H associated with Constant Spring Hemoglobin: preterm and low-birth-weight infant.

The indication for the mode of delivery is obstetric-gynecological and should be shared in a multidisciplinary team including experts in hemoglobinopathies, gynecologists and anesthesiologists, evaluating each case individually.

Strength of the Recommendation: Strong based on weak evidence certainties

Degree of evidence: IIC

Panel agreement: Full agreement

#### 3.3.4. Question No 6 (SCD): Should a Woman with Sickle Cell Disease Who Wishes to Become Pregnant Undergo Specific Checks in Addition to Those Required for Healthy Women [37,48]?

Recommendation No 6: For all women with sickle cell disease who wish to become pregnant, the panel recommends specific evaluations and tests, in addition to what is provided by the Ministerial Decree of 10 September 1998.

**Figure 5:**
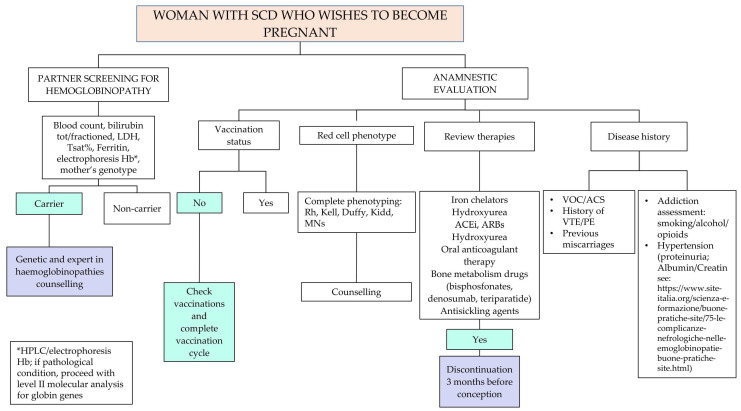
Evaluation and specific tests for women with SCD who wish to become pregnant. LDH: lactate dehydrogenase; Tsat%: transferrin saturation percentage; Hb: hemoglobin; HPLC: high-performance liquid chromatography; ACEi: angiotensin-converting enzyme inhibitor; ARBs: angiotensin receptor blockers; VOC: vaso-occlusive crisis; ACS: acute chest syndrome; VTE: venous thromboembolism; PE: pulmonary embolism.

**Figure 6:**
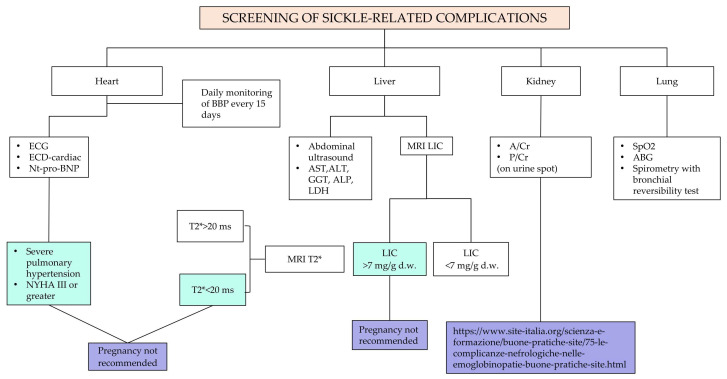
Screening of sickle-related complications in women with SCD: focus on heart, liver, kidney and lung. ECG: electrocardiogram; ECD: cardiac echocolordoppler; Nt-pro-BNP: N-terminal pro–B-type natriuretic peptide; NYHA: New York Heart Association; BBP: brachial blood pressure; T2*: T2 star; ms: millisecond; LIC: liver iron concentration; d.w.: dry weight; A/Cr: albumin-to-creatinine ratio; P/Cr: protein-to-creatinine ratio; SpO2: peripheral oxygen saturation; ABG: arterial blood gas analysis.

**Figure 7:**
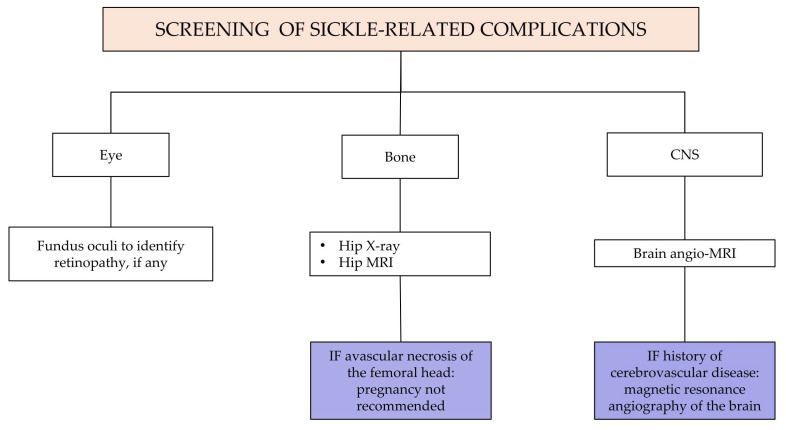
Screening of sickle-related complications in women with SCD: focus on eye, bone and CNS. MRI: magnetic resonance imaging; CNS: nervous.

The descriptive part of Figure 5 is reported below:

- Detailed disease history;
- Partner screening for hemoglobinopathy, determination of the risk of hemoglobinopathy transmission and counseling of the couple by the expert in hemoglobinopathies. Specialist prenatal counseling should be performed prospectively for men and/or women suffering from SCD. The mother’s genotype, if not already available, must be detected. Women with SCD, whose partner is a carrier of a beta-hemoglobin variant (HbS, HbC, HbD, beta thalassemia), have a 50% risk with each pregnancy of giving birth to a child with sickle cell syndrome. These couples should be informed about the aspects of the disease including the possibility of prenatal diagnosis, the associated risks and the possibility of voluntary termination of pregnancy. If the woman is already pregnant at the time of the specialist consultation, counseling must be organized as soon as possible.
- Infectious risk assessment (history and vaccination status, previous isolations of multi-resistant germs);
- Evaluation of the immuno-hematological phenotype: Carry out a collection, as complete as possible, of the patient’s transfusion history and any previous alloimmunizations. Perform complete phenotyping of the Rh system, the Kell system and the antigens of the minor systems (Duffy, Kidd, MNSs). If antibodies for major antibodies are present, perform molecular tests as well. The presence of these antibodies significantly increases the risk of acute or delayed hemolytic reaction that can be associated with hemolytic disease in the fetus or the newborn;
- Review therapies (Table S1): In case of a planned pregnancy, the panel recommends discontinuing pharmacological therapy three months before attempting pregnancy. Accurate counseling on the risk related to the interruption of their treatment should be carried out, followed by the request for patient consent before proceeding. The following drugs should be withdrawn: iron chelators, hydroxyurea (HU, see also Recommendation No 12, note on the use of hydroxyurea in pregnancy) and oral anticoagulants. The patient will have to shift from oral anticoagulant therapy (warfarin, direct oral anticoagulants—DOAC) to therapy with low molecular weight heparin (LMWH). For bone metabolism drugs, discontinuation is indicated three months before for bisphosphonates, while for other therapeutic strategies (e.g., denosumab, teriparatide), an evaluation with the bone metabolism expert is indicated. Concerning antihypertensive molecules such as angiotensin-converting enzyme inhibitors (ACEi) and angiotensin receptor blockers (ARBs), patients need to be switched to other antihypertensive medications not long before pregnancy in agreement with the reference gynecologist/obstetrician;
- Screening for SCD-related chronic organ complications (see Figure 6 and Figure 7):
  - Pulmonary hypertension;
  - Arterial hypertension;
  - Iron overload;
  - Liver disease/cirrhosis/gallbladder disease and spleen evaluation;
  - Sickle-related lung disease;
  - Sickle-related kidney disease;
  - Sickle-related retinopathy;
  - Avascular necrosis of the femoral head or presence of hip replacement;
  - Cerebral events (silent infarctions, strokes, aneurysms, moyamoya disease).

Degree of recommendation: Conditioned based on strong evidence certainties

Degree of evidence: IIIA

Panel agreement: Full agreement

#### 3.3.5. Question No 7 (SCD): In Which Clinical Conditions Is It Advised for a Woman with Sickle Cell Disease Not to Become Pregnant [14,15,49,50,51,52,53,54,55,56,57]?

Recommendation No 7: The panel suggests a woman with sickle cell disease not to become pregnant in the following conditions:

- Obesity (Body Mass Index—BMI > 30);
- Pulmonary hypertension under medical treatment;
- Severe congestive heart failure (New York Heart Association—NYHA class ≥ III);
- Iron overload with organ damage;
- Severe alloimmunization with a non-transfusable profile or previous delayed hemolytic transfusion reaction (DHTR).

Special consideration should be given to women with symptomatic bone necrosis of the femoral head or bone fragility (important to evaluate in relation both to carrying on pregnancy and to the mode of delivery, particularly in case of natural birth).

Degree of recommendation: Conditioned based on strong evidence certainties

Degree of evidence: IIIA

Panel agreement: Full agreement

### 3.4. Management of Pregnancy in Patients with Hemoglobinopathies

#### 3.4.1. Question No 8 (Thalassemia): What Type of Laboratory and Instrumental Follow-Up Is Recommended in Pregnant Women with Thalassemia [12,21,22,38,40,42,58,59,60,61]?

Recommendation No 8: The panel suggests that, in addition to the follow-up planned for pregnant healthy women, in women with thalassemia, an evaluation by the multidisciplinary team (expert in hemoglobinopathies, gynecologist, obstetrician, cardiologist, diabetologist, psychotherapist and other specialists in case of organ complications) should be carried out for a clinical overview and for check-up planning.

The panel, in particular, recommends the following:

- Assessment of cardiac performance: Throughout pregnancy by cardiological examination (if available by a cardiologist with expertise in thalassemia) with ECG and cardiac ECD during the second/third trimester. The literature reports cardiac complications concern women with known cardiac dysfunction (heart failure in hypertrophic cardiopathy in dilated evolution) and symptomatic tachyarrhythmia (mainly of supraventricular type), a minority of which (series of clinical cases) will have a fatal outcome shortly after delivery.
- Endocrinological follow-up planning:
  - Glucose metabolism: Glucose intolerance, gestational diabetes, diabetes. Fasting blood glucose monitoring should be performed monthly from the very beginning of pregnancy. Gestational diabetes screening should be performed at the 24th–28th week of gestation (the panel refers to “Main endocrinological complications in Hemoglobinopathies: good clinical practice by the Società Italiana Talassemie ed Emoglobinopatie”). Diabetic patients who become pregnant require follow-up to ensure adequate glycemic control with monthly monitoring of serum fructosamine levels and a review of medical therapy when necessary.
  - Thyroid function: If euthyroidism is present, thyroid function should be checked monthly. If hypothyroidism is already being treated by replacement therapy, perform monthly monitoring of thyroid function tests (thyroid-stimulating hormone—TSH, Thyroxine—FT4) until the 20th week and perform at least another check between the 26th and the 32nd gestational week. Make timely adjustments to therapy when necessary (the panel refers to “Main endocrinological complications in Hemoglobinopathies: good clinical practice by the Società Italiana Talassemie ed Emoglobinopatie”).
- Infection prophylaxis and vaccinations: Pay attention to even mild signs of infection especially in splenectomized women in order to promptly implement an antibiotic therapy given the high risk of sepsis (e.g., risk of cholecystitis in women with cholelithiasis or urinary tract infections). Perform flu and COVID-19 vaccination (after the 14th week).
- Clinical monitoring of erythropoiesis masses (EMs): The panel reports the presence of sporadic cases of patients in the literature with NTDT who present neurological symptoms from compression related to the increase in EMs during pregnancy. The panel recommends a joint neurological and neurosurgical evaluation and a strict follow-up.
- Monitoring of iron status: Accurate assessment of iron input and monitoring of indirect iron markers (ferritin in women with thalassemia intermedia and transferrin saturation) at least every 3 months.

Degree of recommendation: Conditioned based on moderate evidence certainties

Degree of evidence: IIIB

Panel agreement: Full agreement

#### 3.4.2. Question No 9 (SCD): What Type of Laboratory and Instrumental Follow-Up Is Recommended in Pregnant Women with Sickle Cell Disease [15,49,50,52,53]?

Recommendation No 9: The panel suggests that, in addition to the follow-up planned for pregnancy in healthy women, an evaluation by the multidisciplinary team made up of an expert in hemoglobinopathies, gynecologist, obstetrician, cardiologist, transfusionist, anesthesiologist, neonatologist and psychologist/CLM should be carried out in women with sickle cell disease for a clinical overview and check-up planning.

In particular, the panel makes the following recommendations:

- Informational interview with the patient and her partner on possible complications that may occur during pregnancy;
- Periodic updates to the patient on how to avoid VOCs and precipitating factors (advice to avoid persistent vomiting), verify daily intake of 5 mg folic acid and discontinuation of contraindicated drugs;
- Infection prophylaxis and vaccinations: Pay attention to even mild signs of infection especially in splenectomized women in order to promptly implement an antibiotic therapy given the high risk of sepsis (e.g., risk of cholecystitis in women with cholelithiasis or urinary tract infections). Perform flu and COVID-19 vaccination (after the 14th week);
- Endocrinological follow-up planning as in healthy women.

Degree of recommendation: Conditioned based on moderate evidence certainties

Degree of evidence: IIIB

Panel agreement: Full agreement

#### 3.4.3. Question No 10 (Thalassemia): Do Pregnant Women with Thalassemia Require Changes in the Transfusion Regimen [12,21,40,59,62,63,64,65]?

Recommendation No 10: The panel makes the following recommendations:

- Women with TDT: Maintain a transfusion threshold of Hb 10 g/dl to allow correct fetal development, with monitoring of the blood count at least every 2–3 weeks.
- Women with NTDT: There is no defined pretransfusion Hb cut-off and there is no precise transfusion interval to be respected to ensure correct fetal development. The decision to have a pregnant NTDT woman undergo a blood transfusion must be evaluated based on multiple factors and consultation with the gynecologist/obstetrician and the expert in hemoglobinopathies. Consider starting a regular transfusion regimen in case of symptomatic anemia, worsening of cardiac function or fetal growth restriction, with the same approach valid for transfusion-dependent thalassemia (pretransfusion Hb around 10 g/dL).

Consider the risk of acute anemia secondary to alloimmunization. Maintain multidisciplinary management with the transfusionist, offering the patient extended phenotyping (ABO, Rh, Kell, Duffy, MNSs antigenic systems), indirect Coombs test and, if positive, identification and titration of alloantibodies, and subsequent assignment of units of red blood cells negative for the antigen towards which the antibody has developed. Cases of severe immuno-mediated acute anemia, poor response to high doses of steroids, worsening hemolytic anemia with progressive splenomegaly and hypersplenism requiring postpartum splenectomy in women with NTDT are described in the literature. In critically ill patients with DHTR, the clinical management might require immunotherapy associated with steroids before transfusion.

Degree of recommendation: Strong based on moderate/weak evidence certainties

Degree of evidence: IIB

Panel agreement: Full agreement

#### 3.4.4. Question No 11 (Thalassemia): Does a Pregnant Woman with Thalassemia Require Changes in Iron Chelation Therapy [12,20,21,22,40,42,60,66,67,68,69,70]?

Recommendation No 11 (Table S1): The panel makes the following recommendations:

- DISCONTINUATION of chelation therapy ideally three months before conception. If this is not possible, discontinue as soon as pregnancy is confirmed (positive test), given the potential teratogenic risk of all iron chelators. In the literature, some cases with favorable outcomes have been described in thalassemic women who continued chelation therapy with deferoxamine (up to the 2nd–3rd trimester), deferasirox (up to the 20th week) or deferiprone (up to the 20th week) for even prolonged periods because they were unaware of the ongoing pregnancy;
- EARLY RESUMPTION of chelation therapy during pregnancy (after the 1st trimester) in case of pathological cardiac T2*, systolic-diastolic dysfunction, symptomatic arrhythmias or when the expected benefits outweigh the potential risks for the fetus, ideally with deferoxamine; in complex cases followed by centers with less expertise, a consultation with expert centers of the SITE network is recommended;
- RESUMPTION of chelation therapy early after delivery with the use of deferoxamine;
- USE OF DEFEROXAMINE during breastfeeding;
- CARDIAC/HEPATIC IRON REASSESSMENT as soon as possible (with T2*/LIC).
- In case of cardiac performance alteration or occurrence of cardiac failure, chelation should be considered as a life-saving therapy. In this case, the panel recommends treatment with continuous IV infusion of deferoxamine.

Degree of recommendation: Strong based on moderate/weak evidence certainties

Degree of evidence: IIb

Panel agreement: Full agreement

#### 3.4.5. Question No 12 (SCD): Does a Pregnant Woman with Sickle Cell Disease Require Changes in Background Therapy and/or the Initiation of a Transfusion Regimen [15,51,52,53,62,65,71,72,73,74,75,76,77,78,79,80,81,82]?

**Figure 8:**
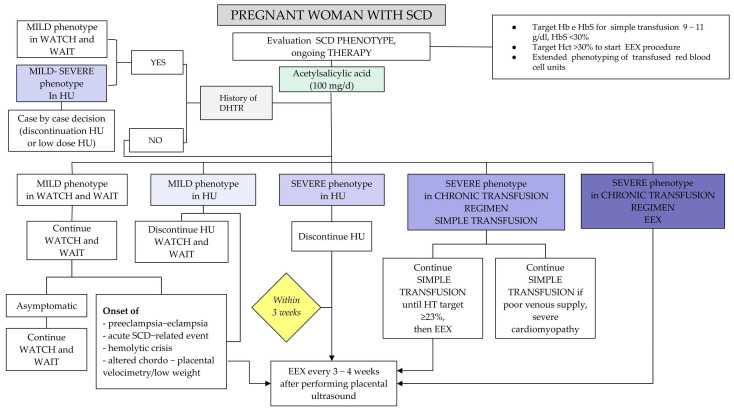
Management of therapy during pregnancy in women with SCD. Hb: hemoglobin; HbS: hemoglobin S; HCT: hematocrit test; EEX; eritro-exchange; DHTR: delayed hemolytic transfusion reaction; HU: hydroxyurea.

Recommendation No 12 (Table S1): For a correct evaluation of the management of therapy during pregnancy, the panel recommends taking into account the phenotype of the disease, the genotype, the therapeutic regimen followed before pregnancy, and some specific aspects. See Figure 8.

The therapeutic choice for the management of SCD during pregnancy, i.e., watch and wait versus transfusion regimen (simple transfusion, manual or automated erythroexchange), must take into account the phenotype of the disease (mild versus severe or presence of organ damage), the therapeutic regimen before pregnancy (watch and wait, hydroxyurea, chronic transfusion therapy) and some specific aspects such as

- Genotype (HbSS versus HbSß and HbSC);
- Previous obstetric history (previous miscarriages and timing of the miscarriages, sickle-related complications in previous pregnancies versus no complications);
- Twin pregnancy (with worse outcomes);
- Transfusion history with particular focus on the presence of alloimmunization (history of alloantibodies or previous delayed hyperhemolytic reaction);
- Previous venous thromboembolic event (VTE).

Regarding the prophylactic transfusion regimen during pregnancy in patients not subjected to chronic transfusion regimen (outside of pregnancy), the lack of prospective, multicenter and randomized studies is reported. The only prospective randomized study dates back to 1988 [83] and evaluates by comparison the prophylactic transfusion regimen versus transfusion on demand for an acute event in 72 patients with SCD: there are no differences in perinatal outcomes and pregnancy-related and obstetrical complications in the two groups; a significant decrease in terms of VOC reduction and SCD-related complications is reported in the treated group.

There is considerable variability regarding the timing of starting the prophylactic transfusion regimen, usually indicated around the 22nd–28th gestational week. In a retrospective cross-sectional study of 46 pregnancies of women with SCD with a severe phenotype, undergoing prophylactic transfusion therapy with early erythroexchange (10.7 + 5.2 weeks of gestation), a significant reduction in SCD-related complications, medical complications of pregnancy and obstetrical complications was observed compared to transfusion regimens that were started between the 22nd and the 28th week of gestation.

If a prophylactic transfusion regimen is indicated, the panel advises to start it as early as possible (ranging between the 10th and the 14th week)

- Notes on transfusion procedure

Consider the risk of acute anemia secondary to alloimmunization or DHTR. Maintain multidisciplinary management with the transfusionist colleague, offering the patient extended phenotyping (ABO, Rh, Kell, Duffy, MNSs antigenic systems), indirect Coombs test and, if positive, identification and titration of alloantibodies, and subsequent assignment of units of red blood cells negative for the antigen towards which the antibody has developed.

The choice between manual and automated exchange depends on several factors. The advantage of manual exchange is that it can always be performed in the acute setting and at the patient’s bedside. Automated exchange requires the following:

- A facility authorized to carry out apheresis procedures and personnel expert in apheresis procedures;
- Adequate vascular access.

- Note on the use of hydroxyurea in pregnancy (Table S1)

The use of hydroxyurea in pregnancy is contraindicated by the technical data sheet.

Several cases of patients (suffering from SCD but also from other pathologies for which the drug is indicated) who have taken hydroxyurea for the entire duration of pregnancy without adverse events on the newborns are described in the literature. The panel identifies the following special contexts. In women with SCD with a severe phenotype who have no other therapeutic options (e.g., patients with alloimmunization or with a history of delayed hemolytic transfusion reaction), the risk of hydroxyurea discontinuation (risk of VOCs, acute chest syndrome—ACS, worsening anemia) might be greater than the risks associated with continuing treatment. This is even more stringent in patients with a history of DHTR or developing DHTR during pregnancy. The panel suggests that for pregnant women with SCD having these characteristics, it may be proposed to maintain hydroxyurea therapy at the lowest effective dose, evaluating case by case, after a joint discussion between an expert in hemoglobinopathies, obstetrician and neonatologist to inform the patient and her partner about the potential risks and benefits for the patient and the fetus in withdrawing/continuing the drug.

Degree of recommendation: Strong based on moderate evidence certainties

Degree of evidence: IIa

Panel agreement: Full agreement

#### 3.4.6. Question No 13 (Thalassemia): Is Supplementation Indicated in Women with Thalassemia [12,27,84,85]?

Recommendation No 13 (Table S1): The panel recommends supplementation with folic acid and vitamin D. The panel recommends no iron supplementation.

Supplementation with folic acid: YES

The recommended dose of folic acid is 5 mg per day, starting during the preconception period (even three months before if the pregnancy is planned) and continuing throughout gestation.

Supplementation with vitamin D: YES

Vitamin D levels should be optimized before conception and maintained in the normal range during gestation, or at least above 30 ng/mL. If vitamin D 25-hydroxyvitamin D (25OHD) < 20 ng/mL, 600,000 IU to be distributed over 1–3 months; if 25OHD < 30 ng/mL and >20 ng/mL, 300,000 IU to be distributed over 1–3 months; continue with a daily maintenance dose of 1000 IU or 100,000 IU per month. The panel recommends monitoring serum calcium and vitamin D levels (first, fourth and eighth month) and refers to the document “Raccomandazioni per il management delle malattie metaboliche dell’osso nelle emoglobinopatie “[Recommendations for the management of bone metabolic diseases in hemoglobinopathies] of the Società Italiana Talassemie ed Emoglobinopatie (SITE)” for therapeutic regimens.

Iron supplementation: NO

We remind that patients with NTDT and TDT may be affected by iron overload; therefore, they do not require specific supplementation.

Degree of recommendation: Strong based on weak evidence certainties

Degree of evidence: IIC

Panel agreement: Full agreement

#### 3.4.7. Question No 14 (SCD): Is Supplementation Indicated in Women with Sickle Cell Disease [15,52,84,86,87,88,89,90]?

Recommendation No 14 (Table S1): The panel recommends supplementation with folic acid and vitamin D. The panel recommends iron supplementation if transferrin saturation (TSAT) < 20%. The panel does not recommend antibiotic prophylaxis.

Supplementation with folic acid: YES

The recommended dose of folic acid is 5 mg per day, which should be maintained throughout pregnancy (ideally also in the pre-conceptional period).

Supplementation with vitamin D: YES

Vitamin D levels should be optimized before conception and maintained in the normal range during gestation, or at least above 30 ng/mL. If vitamin D 25OHD < 20 ng/mL, 600,000 IU to be distributed over 1–3 months; if 25OHD < 30 ng/mL and >20 ng/mL, 300,000 IU to be distributed over 1–3 months; continue with a daily maintenance dose of 1000 IU or 100,000 IU per month. The panel recommends monitoring serum calcium and vitamin D levels (first, fourth and eighth month) and refers to the document “Raccomandazioni per il management delle malattie metaboliche dell’osso nelle emoglobinopatie” [Recommendations for the management of bone metabolic diseases in hemoglobinopathies] of the Società Italiana Talassemie ed Emoglobinopatie (SITE)” for therapeutic regimens.

Iron supplementation: YES, if TSAT < 20%

Iron supplementation (liposomal formulation is to be preferred) is indicated in case of documented iron deficiency or iron deficiency anemia or after evaluation of blood count and iron deposits: transferrin saturation (TSAT) and ferritin. Iron supplementation should be started in case of extreme iron deficiency TSAT < 20%. The duration of iron supplementation will be evaluated periodically.

Penicillin prophylaxis: NO

At present, antibiotic prophylaxis with penicillin is not recommended during pregnancy.

Degree of recommendation: Strong based on weak evidence certainties

Degree of evidence: IIc

Panel agreement: Full agreement

### 3.5. Thrombotic Risk Management During Pregnancy in Patients with Hemoglobinopathies

#### 3.5.1. Question No 15 (Thalassemia): Is Antithrombotic/Thromboembolic Prophylaxis Indicated in Women with Thalassemia? If So, Which One [12,91,92,93,94,95]?

Recommendation No 15 (Table S1): The panel makes the following recommendations:

- Assessment of the individual thrombotic risk with collection of the personal and family history of VTE, personal history of obstetrical complications, recurrent miscarriages, splenectomy, extensive thrombophilia screening (antiphospholipid antibodies, factor V Leiden mutation, Factor II—prothrombin—mutation) and complete anamnestic collection;
- Initiation of antiplatelet prophylaxis with acetylsalicylic acid (ASA) 100 mg daily in post-splenectomy TDT and NTDT women;
- Initiation of antithrombotic prophylaxis with LMWH for 6 weeks in the postpartum period regardless of delivery mode if the patient has NTDT; if the patient has TDT and her delivery was vaginal, LMWH for 7 days, and in case of TDT and cesarean section, LMWH for 6 weeks;
- Initiation of antithrombotic prophylaxis with LMWH in case of miscarriage or voluntary termination of pregnancy;
- Active monitoring of signs and symptoms suggestive of thrombotic complications.

Degree of recommendation: Conditioned based on weak evidence certainties

Degree of evidence: Expert consensus IV

Panel agreement: Full agreement

#### 3.5.2. Question No 16 (SCD): Is Antithrombotic/Thromboembolic Prophylaxis Indicated in Women with Sickle Cell Disease? If So, Which One [14,96,97,98,99,100,101,102,103,104,105,106]?

Recommendation No 16 (Table S1): The panel makes the following recommendations:

- Assessment of the individual thrombotic risk with complete anamnestic collection of previous deep vein thrombosis, hospitalizations for SCD-related complications, obesity, thrombocytosis, isolated thrombocytopenia related to African ethnicity or previous pregnancies, recurrent miscarriages and extensive thrombophilia screening (antiphospholipid antibodies, factor V Leiden mutation, Factor II—prothrombin—mutation);
- Initiation of antiplatelet prophylaxis with ASA 100 mg daily from the 12th week to the 36th week also considering the increased risk of pre-eclampsia/eclampsia;
- Initiation of antithrombotic prophylaxis with LMWH from the 28th week until the 6th week after delivery in women at high thrombotic risk;
- Initiation of antithrombotic prophylaxis with LMWH in case of miscarriage or voluntary termination of pregnancy;
- Initiation of antithrombotic prophylaxis with LMWH in the postpartum period (within the first 12 h after a vaginal delivery and within 24 h after a cesarean section), to be continued for 6 weeks regardless of the type of delivery;
- Initiation of antithrombotic prophylaxis with LMWH during VOCs with hospitalization and continuation until the end of pregnancy;
- History of venous thromboembolism (VTE): full-dose LMWH (enoxaparin 1 mg/kg every 12 h) until 6 weeks postpartum, unless on chronic anticoagulant therapy;
- History of stroke: low-dose anticoagulant in addition to low-dose aspirin as suggested by the Canadian Heart Association for prevention of recurrent stroke;
- Active monitoring of signs and symptoms suggestive of thrombotic complications.

If prothrombotic factors are present in addition to hemoglobinopathy, a combination of antiaggregating and antithrombotic prophylaxis should be considered case by case and after a joint discussion between an expert in hemoglobinopathies, obstetrician and gynecologist for risk–benefit assessment.

Degree of recommendation: Strong based on weak evidence certainties

Degree of evidence: IIC

Panel agreement: Full agreement

### 3.6. Management of Pregnancy-Related Complications in Patients with Hemoglobinopathies

#### 3.6.1. Question No 17 (Thalassemia): Are There Any Obstetrical/Gynecological Complications in Pregnant Women with Thalassemia Compared to the Healthy Population and If So, How Should They Be Managed [21,22,40,59,107,108,109,110]?

Recommendation No 17 (Table S2): Given the presence in the literature of NTDT patients reporting low birth weight (LBW) and an increased frequency of intrauterine growth restriction (IUGR), the panel recommends to discuss performing an ultrasound with cord/placental velocimetry with the referring obstetrician. Obstetrical complications specific to pregnant women suffering from thalassemia are reported (in a slightly higher percentage than the general population with non-statistically significant differences):

- Abruptio placentae;
- Placental ischemia;
- Placenta previa;
- Shoulder dystocia (often due to cephalo-pelvic disproportion);
- Preterm birth;
- LBW.

For the management of obstetrical/gynecological complications in women with thalassemia, the panel refers to the obstetrical/gynecological guidelines available in the literature (https://www.sigo.it/linee-guida/nazionali/, accessed on 31 October 2024).

Degree of recommendation: Conditioned based on weak evidence certainties

Degree of evidence: Expert consensus IV

Panel agreement: Full agreement

#### 3.6.2. Question No 18 (Sickle Cell Disease): Are There Any Obstetrical/Gynecological Complications Specific to Pregnant Women with Sickle Cell Disease Compared to Healthy Population and If So, How Should They Be Managed [53,111,112,113,114,115,116,117,118,119,120]?

Recommendation No 18: (Table S2) For the management of obstetrical/gynecological complications, the panel recommends:

- Start ASA therapy at the dose of 100 mg per day for all pregnant women from the 12th to the 36th week of gestation as a prophylaxis for pre-eclampsia (see Figure 8);
- Request flowmetric ultrasound in light of the presence of sickle-related placental pathologies and the increased risk of IUGR and LBW;
- Perform a urine test and urine culture monthly after the beginning of pregnancy and repeat them whenever infectious symptoms are present. Although in the absence of strong evidence, recent British guidelines suggest resuming or starting daily antibiotic prophylaxis, the panel recommends treating women who develop infectious signs and symptoms associated with urinary tract infections (fever, dysuria and increased specific inflammation indices) with empirical antibiotic therapy until the result of the antibiogram. In the case of asymptomatic bacteriuria, urine culture is indicated and possible antibiotic treatment should be considered;
- Home monitoring of blood pressure every 15 days and at each medical visit to evaluate its trend (it is important to know blood pressure values before pregnancy and at the beginning of pregnancy, before the physiological drop in blood pressure by the end of the first trimester of pregnancy which could hide underlying chronic hypertension.) Pressure values >120/70 mmHg or increased systolic or diastolic pressure >20% requires close monitoring to exclude a pregnancy-related hypertensive disorder. If blood pressure values ≥130/80 mmHg appear in at least two separate measurements, consider the diagnosis of pregnancy-related hypertensive disorder and initiate antihypertensive drug therapy in agreement with the referring obstetrician. The woman must be informed of the risks, the importance of home blood pressure monitoring and the “warning” symptoms (headache, edema, diuresis reduction, excessive weight gain, scotomas, “bar” epigastric pain). Whenever hypertension is detected during pregnancy, in the absence of proteinuria or other relevant symptoms, all laboratory tests that evaluate the presence of the other signs of organ dysfunction should be performed, namely blood count, creatinine, uric acid and transaminase, in order to evaluate the evolution of gestational hypertension towards pre-eclampsia. Women with SCD and proteinuria or pre-existing renal damage may require more frequent monitoring, which should be discussed and shared with the multidisciplinary team that manages high-risk pregnancies (an expert in SCD, gynecologist expert in high-risk pregnancies, nephrologist). In the absence of 24 h proteinuria or the PrCr ratio, the dipstick allows a reasonable assessment of real proteinuria, in particular when values are higher than “2+” (higher than 1 gr/L) (AIPE recommendations). For further details, refer to the document “Le complicanze nefrologiche nelle emoglobinopatie. Buone pratiche della Società Italiana Talassemie ed Emoglobinopatie (SITE)” [Nephrological complications in emoglobinopathies. Good practice by the Società Italiana Talassemie ed Emoglobinopatie (SITE)]. If the woman is already undergoing therapy with antihypertensive drugs that are not allowed during pregnancy, the multidisciplinary team must organize an antihypertensive therapeutic plan with a better safety profile during pregnancy;
- Offer erythroexchange to all pregnant women who develop pre-eclampsia or eclampsia (see Figure 8);
- Pre-eclampsia, eclampsia, hemolysis, elevated liver enzymes, low platelet count (HELLP) syndrome. Nonsteroidal anti-inflammatory drugs (NSAIDs) for pain should be avoided in women with pre-eclampsia who have just given birth unless they fail to respond to other painkillers (Recommendations of the Associazione Italiana Pre-eclampsia, AIPE 2020).

Degree of recommendation: Strong based on weak evidence certainties

Degree of evidence: IIC

Panel agreement: Full agreement

### 3.7. Management of Acute Complications During Pregnancy Related to Sickle Cell Disease

#### Question No 19 (SCD): How Should Acute Events Related to Sickle Cell Disease in Pregnant Women Be Managed [14,15,110,119,120,121,122,123,124,125,126,127,128,129,130,131,132,133,134,135]?

Recommendation No 19 (Tables S1 and S2): The panel pinpointed the following acute conditions related to SCD during pregnancy that require rapid identification and intensive treatment:

- VOC;
- ACS;
- STROKE;
- VTE;
- Acute renal failure/renal infarction;
- Sepsis (2–13 times higher risk of infectious complications, including sepsis https://www.sccm.org/Clinical-Resources/Guidelines/Guidelines/Surviving-Sepsis-Guidelines-2021, accessed on 31 October 2024).

If the patient presents one or more of the above-mentioned complications during pregnancy, the panel advises to consider her as having a severe phenotype (if she did not have it previously). She must therefore start chronic transfusion treatment (see Figure 8), maintaining a close interaction with obstetricians for fetal monitoring.

- VOC (Figure 9)

A total of 27–50% of women with SCD present a painful crisis (VOC) during pregnancy, more often in the third trimester. VOC events may be triggered by conditions of hypoxia/dehydration, such as infections, fever, hypovolemia (e.g., vomiting, diarrhea, profuse sweating), prolonged immobility, extreme temperatures and physical or psychological stress; they can also be triggered by traumas or drugs such as anesthetics or cortisone. Please note that the approach to a pregnant woman must be multidisciplinary and involve experts in hemoglobinopathies, obstetricians, gynecologists and anesthesiologists.

For triage and management of acute events, please refer to SITE guidelines.

**Figure 9:**
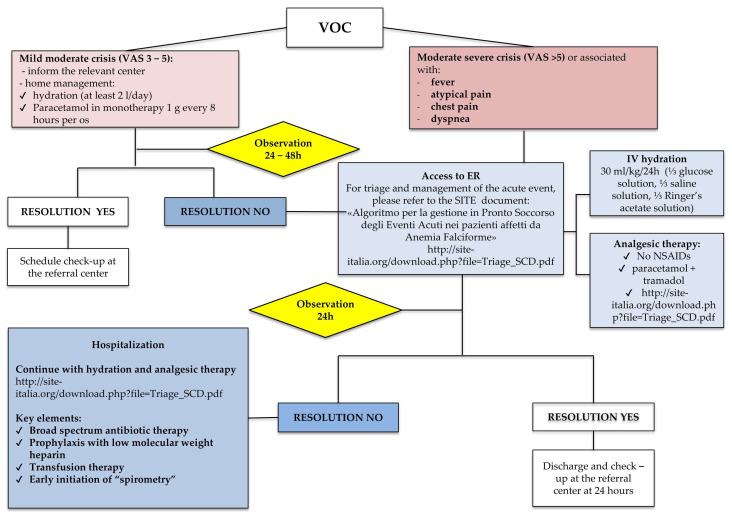
Management of VOC in pregnant woman with SCD. VOC: vaso-occlusive crisis; VAS: visual analogue scale; ER: emergency room; IV: intravenous; NSAIDs: non-steroidal anti-inflammatory drugs; h: hours.

The treatment of VOCs includes the following (Figure 9):

- Intravenous hydration: 30 mL/kg/24 h (⅓ glucose solution, ⅓ saline solution, ⅓ Ringer’s acetate solution);
- Analgesic therapy: Administration within 30 min of admission to the hospital, with reassessment every 30 min and pain reduction (Visual Analogue Scale—VAS) within 60 min. Analgesic therapy should begin with intravenous administration of paracetamol at a maximum of 1 g every 8 h. If the pain is not controlled, use multimodal analgesia, combining paracetamol with opioids in continuous IV infusion, buccal or nasal fentanyl as a pain-breaking drug. Pethidine should be avoided for the risk of toxicity or convulsions. Multimodal analgesia is based on the administration of drugs with different pharmacological mechanisms of action, controlling pain of various origins (e.g., vascular, somatic and neuropathic). This maximizes analgesia and minimizes adverse side effects.
- Early start of respiratory rehabilitation program for the recovery of diaphragm mobility and expiratory phase.

Transfusion therapy

- Transfusion of erythrocyte concentrates (ECs) is the choice whenever Hb ≤ or equal to 7 g/dL, in the absence of an immuno-hematological picture suggestive of hyperhemolytic syndrome and/or based on the severity of the clinical picture; Hb 8–9 g/dL, manual erythroexchange is indicated; Hb > 10–11 g/dL, manual or automatized erythroexchange is indicated. The impact of the transfusion approach on the percentage of HbS has to be determined at least one time before patient discharge.
- Manual or automated procedure of erythroexchange (EEX): It is performed to rapidly obtain HBs levels < 30% and is preferred to classic transfusion due to the reduced risks of volumetric overload, iron overload and hyperviscosity. Placental ultrasound evaluation is indicated before the session to exclude placental abruption, which would be a contraindication to the automated erythroexchange procedure.

- ACS (Figure 10)

ACS occurs in 7–20% of pregnant women suffering from SCD and is a major cause of mortality. ACS is most frequent in the 3rd trimester of pregnancy and in the 48–72 h following delivery.

The treatment of ACS is similar to that of VOCs, with particular attention to oxygenation with periodic blood gas analysis checks.

- VTE (Figure 10)

The risk of thromboembolism in pregnant women suffering from SCD is 1.5 times higher than in the general population. The prevalence of VTE in pregnant women with complications like ACS and VOC is then 3.5 times higher than in women with no disease-related complications. The prevalence of pulmonary embolism (PE) in pregnant women with SCD is 2.5 times higher than the occurrence of venous thromboses. The major thromboembolic events occur in the 3rd trimester of pregnancy and especially in the postpartum period. PE should be suspected in any woman presenting with chest pain and acute respiratory failure.

**Figure 10:**
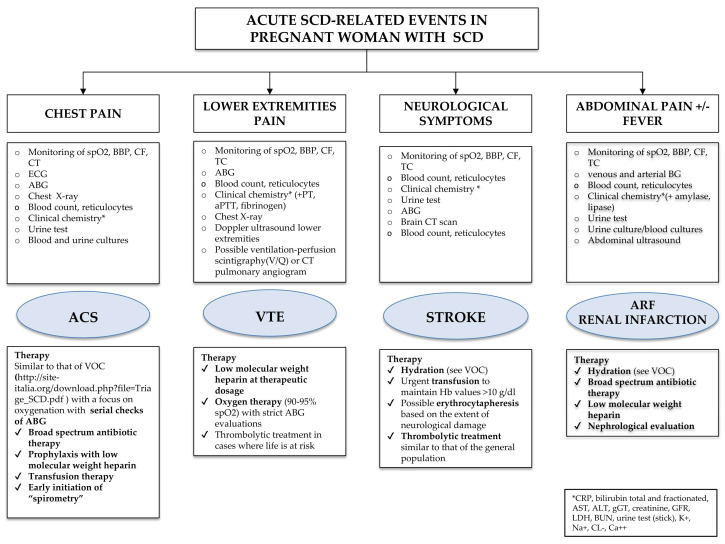
Management of acute SCD-related events in pregnant women with SCD. SpO2: peripheral oxygen saturation; BBP: brachial blood pressure; CF: cardiac frequency; CT: corporeal temperature; ECG: electrocardiogram; ABG: arterial blood gas analysis; ACS: acute chest syndrome; VOC: vaso-occlusive crisis; PT: prothrombin time; aPTT: activated partial thromboplastin time; V/Q: ventilation/perfusion; CT: computed tomography; VTE: venous thromboembolism; AFR: acute renal failure; CRP: c-reactive protein test; AST: aspartate transaminase; ALT alanine transaminase; GGT: gamma-glutamyl transferase; Ca^2+^: calcium.

In the case of VTE, consider the following:

- High d-dimer values are not decisive as they have already increased during SCD.
- Echocolor Doppler of lower extremities:
  ○ If positive, no further investigations are necessary; start LMWH at an anticoagulant dose;
  ○ If negative, but there is clinical suspicion of pulmonary embolism remains significant, use a CT pulmonary angiogram (www.acr.org, accessed on 31 October 2024) according to clinical judgment based on the availability of the equipment, the patient’s informed consent and comorbidities (kidney failure, allergy to contrast media). In the case of PE, treatment with LMWH at therapeutic dosage is preferable to unfractionated heparin and oxygen therapy with thorough assessments of arterial blood gas analysis. Thrombolytic treatments should be reserved for patients at risk of life (hemodynamic instability, www.escardio.org, accessed on 31 October 2024).

- STROKE (Figure 10)

Acute neurological manifestations that may affect a pregnant woman with SCD are as follows: cerebral infarction with ischemia (stroke), recurrent transient ischemic attacks (TIA), intracranial hemorrhage, cognitive impairment related to silent cerebral infarctions.

Acute stroke, whether hemorrhagic or ischemic, should be taken into consideration for any woman who suddenly shows neurological signs and symptoms (e.g., constant headache, aphasia, dysarthria, signs of hiatus).

Treatment in case of stroke involves circulatory support with hydration (similar to that during VOC) and urgent transfusion to maintain Hb values > 10 g/dL and limit brain damage. In patients with SCD, thrombolytic treatment similar to that used for the general population is indicated. In the 5–24 h following the acute event, consider EEX for an HbS target of 15–20%.

- ACUTE KIDNEY FAILURE OR KIDNEY INFARCTION (Figure 10)

During pregnancy, acute kidney failure (from pyelonephritis or papillary necrosis) may arise during VOC or complicate a VOC frequently in the course of ACS or multi-organ failure (MOF). For details, the panel refers to the document “Le complicanze nefrologiche nelle emoglobinopatie. Buone pratiche della Società Italiana Talassemie ed Emoglobinopatie (SITE)” [“Nephrological complications in hemoglobinopathies. Good practice of the Società Italiana Talassemie ed Emoglobinopatie (SITE)”]

Degree of recommendation: Strong based on weak evidence certainties

Degree of evidence: IIC

Panel agreement: Full agreement

### 3.8. Childbirth: Pre-, Intra-, Postpartum in Women with Hemoglobinopathy

#### 3.8.1. Question No 20 (Thalassemia): Do Women with Thalassemia Require Management of the Stages of Childbirth Different from That of Healthy Women [21,22,40,42,109]?

Recommendation No 20: The panel suggests that the indication for the delivery mode is obstetric-gynecological, considering the peculiar characteristics of the thalassemia patient and evaluating case by case (for example, cephalo-pelvic disproportion). With regard to anesthesia/sedation, the panel suggests to bring the following critical issues to the attention of the anesthesiologist/resuscitator:

- Maxillo-facial deformities that could make intubation and maintenance of airway patency more complicated;
- Severe spinal deformities (for example scoliosis), osteoporosis causing somatic collapses or reduction in inter-somatic spaces or presence of vertebral masses due to extramedullary hematopoiesis (EMH).

Degree of recommendation: Conditioned based on weak evidence certainties

Degree of evidence: Expert consensus IV

Panel agreement: Full agreement

#### 3.8.2. Question No 21 (SCD): Do Women with Sickle Cell Disease Require Management of the Stages of Childbirth Different from That of Healthy Women [14,15,52,53,117,136]?

Recommendation No 21 (Table S1): The panel makes the following recommendations:

- Schedule delivery, when possible, in facilities where acute SCD-related complications can be managed with monitoring of peripheral oxygen saturation (to be maintained at 90–94%) and continuous fetal cardiotocographic monitoring;
- Avoid prolonging labor in the periods of dilation and expulsion (no more than 10 h);
- Maintain the woman in labor at a constant temperature;
- Ensure adequate hydration with at least 30–50 mL/kg/day (⅓ 0.9% saline, ⅓ 5% glucose solution, ⅓ Ringer’s acetate solution while monitoring average brachial blood pressure at a target of 70 mmHg);
- Ensure adequate pain control;
- Ensure, before delivery, a target Hb between 9 and 11 g/dL and HbS < 30% by means of simple transfusion or EEX (manual or automated) to be performed no more than one week from delivery (see https://www.site-italia.org/storage/site/article/pdf/36/1-Collana_scientifica_SITE_n.2_2014.pdf, accessed on 31 October 2024);
- Maintain adequate hydration in the postpartum period (30 mL/kg/day) and continuous monitoring of peripheral oxygen saturation;
- Start early antibiotic therapy if signs of infection appear.

There are no randomized controlled trials indicating a clear timing of delivery. It is appropriate to consider planning delivery at the 37th week for genotypes SS, Sβ0 and other genotypes with severe clinical phenotypes. Please refer to the guidelines of the British Royal College of Obstetricians and Gynaecologists (https://www.rcog.org.uk/guidance/browse-all-guidance/green-top-guidelines/, accessed on 31 October 2024). The indication for the delivery mode is obstetric-gynecological, taking into account the peculiar characteristics of the sickle cell patient and evaluating each individual case. In vaginal delivery, previous avascular necrosis of the femoral head or the presence of a hip replacement that may limit rotation and abduction movements must be taken into account. For cesarean delivery, the higher risk of infectious complications and VTE in women with SCD compared to healthy women must be considered. In addition, in women with SCD, there is an increased risk of complications such as intrauterine death and preterm delivery (due to placental abruption or placental insufficiency). Therefore, whenever possible, the delivery should be scheduled in facilities equipped with a Neonatal Intensive Care Unit (NICU).

**Labor**

Labor is a trigger factor for VOC. Therefore, it is essential to avoid prolonged labor, which should not be longer than 10 h as it is associated with a greater risk of painful crises and/or ACS. It is necessary to ensure an adequately heated environment, adequate hydration by infusion (30–50 mL/Kg/day iv, ⅓ of 0.9% saline, ⅓ of 5% glucose solution, ⅓ of Ringer’s acetate solution, while monitoring average brachial blood pressure with a target of 70 mmHg) and adequate oxygenation of the patient (monitor pulse oximetry and use oxygen mask during labor and delivery for a target of SaO_2_ 90–94%). Perform continuous fetal cardiotocographic monitoring to document fetal distress situations that may require immediate delivery and ensure good pain control.

**Pain relief therapy**

During the third trimester of pregnancy, pain relief therapy should be discussed with the anesthesiologist. Pain relief therapy during labor should be agreed upon with the anesthesiologist/consulting pain therapist. At the time of delivery, a value of Hb between 9 and 11 g/dL with HbS < 30% is recommended. In the case of a cesarean section, if the patient is not already on a chronic transfusion regimen, there is an indication for pre-operative transfusion (manual or automated erythroexchange or simple transfusion in relation to the Hb value), as it is a surgical procedure (see https://www.site-italia.org/storage/site/article/pdf/36/1-Collana_scientifica_SITE_n.2_2014.pdf, accessed on 31 October 2024). The transfusion procedure must be performed no more than one week before the scheduled operation. Transfusion can also be performed after delivery based on losses as indicated by the obstetrician.

**Postpartum**

In the postpartum period, there is a high risk of VOCs, infectious complications (e.g., urinary tract infections and pneumonia) and/or thrombotic complications (e.g., VTE); therefore, adequate hydration (30 mL/Kg/day iv, ⅓ of 0.9% saline, ⅓ of 5% glucose solution ⅓ of Ringer’ Acetate solution) and peripheral monitoring of SaO_2_ (target of peripheral oxygen saturation -spO2- 90–94%) are recommended. It is useful to encourage the patient to early mobilization, recommend compression elastic stockings and start prophylaxis with low molecular weight heparin during hospitalization (2 h after vaginal birth and 8 h after cesarean section), which is to be continued for 6 weeks. In the case of VOCs, nonsteroidal anti-inflammatory drugs can be used in the postpartum period and during breastfeeding.

Degree of recommendation: Conditioned based on strong evidence certainties

Degree of evidence: IIIa

Panel agreement: Full agreement

### 3.9. Puerperium in Women with Hemoglobinopathies

#### 3.9.1. Question No 22 (Thalassemia, SCD): Is Breastfeeding Recommended in Women with Thalassemia and Sickle Cell Disease?

Recommendation No 22: The panel also recommends breastfeeding in patients with thalassemia and SCD, except for those with HIV, detectable HCV RNA and/or Hepatitis B surface antigen (HbsAg) positivity.

Degree of recommendation: Conditioned based on weak evidence certainties

Degree of evidence: Expert consensus IV

Panel agreement: Full agreement

#### 3.9.2. Question No 23 Are There Any Preventive Strategy to Be Followed in the First 3–6 Months After Delivery [12,21,22,40,73,79,107,109,137,138,139,140]?

Recommendation No 23 (Table S1): For women with hemoglobinopathy, the panel recommends the following:

- Carry on with vitamin D supplementation that started during pregnancy and maintain it throughout the breastfeeding period;
- Do not start or reintroduce bisphosphonate therapy during breastfeeding;
- Reassess thyroid function with TSH and adjust therapy with Levothyroxine;
- If gestational diabetes is observed, the patient should be managed in the same way as non-hemoglobinopathic patients. For women with diabetes, the use of metformin is not advised, while there are no contraindications to the use of insulin;
- In women with TDT: Use iron chelation therapy with deferoxamine during breastfeeding. Periodically reassess the duration/continuation of breastfeeding based on the need for intensive iron chelation and the patient’s clinical conditions (e.g., bone weakening, cardiopathy);
- In women with SCD: Maintain the transfusion strategy implemented during pregnancy and gradually reintroduce HU until the pre-pregnancy dosage is reached. Consider the discontinuation of the transfusion regimen at least 1 month from the start of HU and within 3 months of delivery. If VOC is mild, start analgesic treatment with paracetamol and ibuprofen at a standard dosage; if VOC is moderate/severe, refer to Figure 8 and Figure 9; we also recommend the discontinuation of breastfeeding.

During breastfeeding, the resumption of the contraceptive therapy previously used by the patient is not contraindicated.

Degree of recommendation: Conditioned based on weak evidence certainties

Degree of evidence: Expert consensus IV

Panel agreement: Full agreement

## 4. Discussion

Thalassemic syndrome and SCD are hereditary red cell disorders with a negative impact on patients’ quality of life that are distributed worldwide. Currently, limited good clinical practice recommendations are available for both disorders, making the decision-making process challenging for physicians from less expert centers [110]. In addition, the increased life expectancy and the introduction of screening programs associated with intensive medical treatment have beneficially impacted patients’ life expectancy with a reduction/delay in severe organ damage [5,6,7,8,9,10,11]. Thus, the desire for pregnancy becomes real for women with either thalassemia or SCD, requiring a multidisciplinary team of health professionals [12,13,14,15]. In women with thalassemia, typical pregnancy issues are more related to the fertility rate, with some special conditions such as iron overload cardiomyopathy. However, maternal mortality and morbidity among women with SCD are still higher when compared to a matched healthy population, suggesting the requirement for close follow-up and intense medical treatment during pregnancy and after delivery to ameliorate maternal and fetal outcomes in SCD women [78,141]. This multidisciplinary approach might even turn out to be transdisciplinary when ethnocultural diversity may contribute to increased maternal mortality during pregnancy, as observed in a French cohort of recent female migrants with SCD [18,19]. It is from this perspective that we consider it crucial to add a section on pregnancy from a medical anthropologic angle. Indeed, pregnancy can be considered a space for multicultural encounters, using relationship moments as tools for optimal birth support. We believe that HCPs need to be aware of, or ideally trained in, “not medical needs” to overcome the risk of reductive stereotypes. This might optimize both the communication and negotiation phases, which are part of the clinical management of all patients, but it is mandatory during pregnancy. Last but not least, our extensive experience in developing national strategies to prevent hemoglobinopathies has shown that legislative interventions may be necessary in endemic areas for hemoglobinopathies [142]. Thus, in 1992, the Italian Ministry of Health answered with a law, offering free tests and counseling for hemoglobinopathies to couples attending pregnancy or to expectant couples at the first obstetric/gynecologic evaluation. Thus, the improvement of screening strategies should be considered a priority in endemic areas for hemoglobinopathies and in areas with a high migration index from endemic areas for hemoglobinopathies [142].

In conclusion, the present GP document on pregnancy in patients with either thalassemia or SCD might be a useful tool in daily practice, especially with the inclusion of newly created, user-friendly flowcharts. We plan to disseminate this GP document throughout the SITE network to reach both less expert centers and patient advocacy groups. We believe that this strategy might reduce the burden of pregnancy in these patient populations. Future studies will be designed to evaluate the impact of this GP document on maternal and fetal outcomes in women with ether thalassemia or SCD within the SITE network in 3 years.

## Data Availability

Not applicable.

## Supplementary Materials

The following supporting information can be downloaded at https://www.mdpi.com/article/10.3390/jcm14030948/s1, Table S1. Recommended ad hoc changes in standard treatment of women with either thalassemia or sickle cell disease during the various phases of pregnancy. Table S2. Clinical complications of pregnancy in women with either thalassemia or sickle cell disease.
