# Supplementary material for: Thalassemias and Sickle Cell Diseases in Pregnancy: SITE Good Practice
Source: J Clin Med. 2025 Feb 1;14(3):948. doi: 10.3390/jcm14030948 (PMC11818879; doi:10.3390/jcm14030948)
Supplement: Supplementary file 1 [file jcm-14-00948-s001.zip › jcm-3372270-supplementary.pdf]

Table S1. Recommended *ad hoc* changes in standard treatment of women with either thalassemia or sickle cell disease during the various phase of pregnancy				
	**Pre-conception stage (three months before attempting pregnancy)**	**Early gestation**	**Mid-term gestation and labor**	**Brest-feeding**
TDT- NTDT	STOP<br>• iron chelation therapy<br>• agent(s) targeting erythropoiesis such as Luspatercept<br>• bisphosphonates (for other bone therapy consult with bone metabolism expert)<br>• warfarin and DOAC and start LMWH<br>• ACEi and ARBs and start antihypertensive medications in agreement with the reference gynecologist/obstetrician	START<br>• ASA in splenectomized patients<br><br>MAITAIN<br>• folic acid and vitamin D supplementation	STOP<br>• ASA at 36 weeks of gestation<br><br>START<br>• prophylaxis with LMWH for 6 weeks in the postpartum period if case of NTDT (regardless of delivery mode)<br>• prophylaxis with LMWH for 6 weeks in the postpartum period if case of TDT and cesarean delivery<br>• prophylaxis with LMWH for 7 days in case of TDT and vaginal delivery	MAINTAIN<br>• folic acid and vitamin D supplementation<br><br>START<br>• contraception<br>• based on CC: iron chelation therapy with deferoxamine (see recommendation No 11 and 23)<br><br>STOP<br>• bisphosphonates (for other bone therapy consult with bone metabolism expert)
SCD	STOP<br>• HU<br>• iron chelation therapy<br>• antisickling agents<br>• crizanlizumab<br>• bisphosphonates (for other bone therapy consult with bone metabolism expert)	START<br>• transfusion regimen based on the clinical phenotype (see Figure 8)<br>• ASA from 12 weeks<br>• Antithrombotic<br>• low-dose anticoagulant in addition to low-dose	STOP<br>• ASA at 36 weeks of gestation<br><br>START<br>• LMWH prophylaxis from 28thweek until the 6 weeks after delivery in	MAINTAIN<br>• folic acid and vitamin D supplementation (see also Figure 8-9)<br><br>START<br>• contraception<br>• based on CC: iron chelation therapy with

	• stop warfarin and DOAC and start LMWH<br>• stop ACEi and ARBs and start antihypertensive medications in agreement with the reference gynecologist/obstetrician	aspirin, if history of stroke (see also Figure)<br><br>MAITAIN<br>• folic acid and vitamin D supplementation<br>• HU at the lowest effective dose in case of women with SCD with severe phenotype and with no other therapeutic options (see Note on HU Recommendation No 12)	women at high thrombotic risk<br>• LMWH prophylaxis in the postpartum period (within the first 12 hours after a vaginal delivery and within 24 hours after a cesarean section), to be continued for 6 weeks regardless of the type of delivery<br><br>Labor<br>Ensure:<br>• adequate hydration<br>• adequate pain control<br>• a target Hb between 9 and 11 g/dl and HbS < 30% by means of simple transfusion or EEX (manual or automated) to be performed no more than one week from delivery<br><br>Start early antibiotic therapy if signs of infection appear	deferoxamine (see recommendation No 23)<br><br>STOP<br>• bisphosphonates (for other bone therapy consult with bone metabolism expert)<br><br>NOTE:<br>Consider discontinuation of the transfusion regimen at least 1 month from the start of HU and within 3 months of delivery. If VOC see Figure 8-9 and discontinue breastfeeding

TDT: transfusion dependent thalassemia; NTDT: non-transfusion dependent thalassemia; SCD: sickle cell disease; DOAC: direct oral anticoagulants; LMWH: low molecular weight heparin; ACEi: angiotensin converting enzyme; ARBs: inhibitor angiotensin receptor blockers; HU: hydroxyurea; Hb: hemoglobin; ASA: acetylsalicylic acid; VOC: vaso-occlusive crisis; VTE: venous thromboembolic events; T-sat: transferrin saturation; EMs: erythropoiesis masses; HbS: hemoglobin S; EEX: erythroexchange; CC: cardiac status

Table S2. Clinical Complications of pregnancy in women with either thalassemia or sickle cell disease			
	**Complications related to pregnancy**	**Complication of pregnancy**	**Complications related to the hemoglobinopathy**
TDT- NTDT	Deep thrombotic events in splenectomized patients	Abruptio placentae<br>Placental ischemia<br>Placenta previa<br>Shoulder dystocia<br>Preterm birth<br>Low weight birth newborn<br>Intrauterine growth restriction	Cardiac dysfunction<br>Symptomatic tachyarrhythmia<br>Increase of EMs
SCD	Recurrent urinary infections<br>Pyelonephritis<br>Deep thrombotic events	Miscarriages<br>stillbirths<br>Ischemic placental disease<br>Pre-eclampsia<br>Eclampsia<br>HELLP syndrome<br>Intrauterine growth restriction<br>Low weight birth newborn	Acute vaso-occlusive crisis<br>Acute chest syndrome<br>Acute hemolytic crisis<br>Erythroid aplastic crisis<br>DHTR

TDT: transfusion dependent thalassemia; NTDT: non-transfusion dependent thalassemia; SCD: sickle cell disease; HELLP: Preeclampsia, Eclampsia, Hemolysis, Elevated Liver enzymes, Low Platelet count; EMs: erythropoiesis masses; DHTR: delayed hemolytic transfusion reaction
